# Review and Evaluation of Mindfulness-Based iPhone Apps

**DOI:** 10.2196/mhealth.4328

**Published:** 2015-08-19

**Authors:** Madhavan Mani, David J Kavanagh, Leanne Hides, Stoyan R Stoyanov

**Affiliations:** ^1^ Institute of Health & Biomedical Innovation School of Psychology and Counselling Queensland University of Technology Kelvin Grove Australia

**Keywords:** mindfulness, mindfulness-based mobile apps, mobile health (mHealth), mental health

## Abstract

**Background:**

There is growing evidence for the positive impact of mindfulness on wellbeing. Mindfulness-based mobile apps may have potential as an alternative delivery medium for training. While there are hundreds of such apps, there is little information on their quality.

**Objective:**

This study aimed to conduct a systematic review of mindfulness-based iPhone mobile apps and to evaluate their quality using a recently-developed expert rating scale, the Mobile Application Rating Scale (MARS). It also aimed to describe features of selected high-quality mindfulness apps.

**Methods:**

A search for “mindfulness” was conducted in iTunes and Google Apps Marketplace. Apps that provided mindfulness training and education were included. Those containing only reminders, timers or guided meditation tracks were excluded. An expert rater reviewed and rated app quality using the MARS engagement, functionality, visual aesthetics, information quality and subjective quality subscales. A second rater provided MARS ratings on 30% of the apps for inter-rater reliability purposes.

**Results:**

The “mindfulness” search identified 700 apps. However, 94 were duplicates, 6 were not accessible and 40 were not in English. Of the remaining 560, 23 apps met inclusion criteria and were reviewed. The median MARS score was 3.2 (out of 5.0), which exceeded the minimum acceptable score (3.0). The Headspace app had the highest average score (4.0), followed by Smiling Mind (3.7), iMindfulness (3.5) and Mindfulness Daily (3.5). There was a high level of inter-rater reliability between the two MARS raters.

**Conclusions:**

Though many apps claim to be mindfulness-related, most were guided meditation apps, timers, or reminders. Very few had high ratings on the MARS subscales of visual aesthetics, engagement, functionality or information quality. Little evidence is available on the efficacy of the apps in developing mindfulness.

## Introduction

### Background

Mindfulness has grown in popularity in the last two decades, and there is growing evidence for its positive impact on well-being [1,2]. Many different perspectives of mindfulness have evolved over this period. An influential definition by Jon Kabat-Zinn is that mindfulness is “paying attention on purpose, in the present moment, and non-judgmentally to the unfolding of experience moment by moment” (p 145 [3]). Mindfulness is seen as a skill that can be developed through practice. The benefits of present-centered attention and acceptance of experience that can be achieved through mindfulness include enhanced awareness, greater self-regulation, greater openness and acceptance to experiences, and the development of new perspectives on the context and content of information [4]. This contrasts with mindlessness, where an individual’s attention is focused on past experiences and concerns about the future rather than on the present moment [5].

Accordingly, mindfulness has been found to have beneficial psychological, somatic, behavioral, and interpersonal effects [6], developing tolerance, acceptance, patience, trust, openness, gentleness, generosity, empathy, gratitude, and loving-kindness, each of which is relevant to the personal recovery of people with mental disorders, as well as to positive well-being in general [2]. Mindfulness has also been found to reduce psychological distress and optimize psychological functioning in young people [7]. There is growing evidence for the efficacy of mindfulness-based programs in promoting well-being [8], reducing depression [9], and preventing relapse in depression [10].

While mindfulness can be an effective tool for improving health and psychological well-being, finding an effective mindfulness delivery medium that can reach a wider audience remains a challenge.

### Apps for Mental Health

The global prevalence and burden of mental disorders is substantial, and delivering mental health services effectively to millions in need remains a challenge [11]. While Web-based interventions are gaining empirical support [12], mobile interventions are still in their infancy [13]. Mobile health (mHealth) is an emerging field that uses wireless technologies such as mobile phones and other devices in health practice. The advent of apps has created new opportunities. Smartphones can keep the user connected to the Internet at all times. Smartphones and apps provide computing facility comparable to personal computers and software with the advantage of mobility.

Smartphone use is growing rapidly [14], and smartphones now account for 25% of total Web usage. A recent Australian study [15] reported that 88% of its survey respondents use websites or apps on their mobile phone and predicted that 92% of respondents would own a smartphone by October 2015. Global mobile app downloads are expected to reach 269 billion by 2017 [16]. Smartphone usage by young people is particularly high: The Australian Communications and Media Authority reported that in May 2013, 89% of people aged 18-24 years had a smartphone and 83% of this age group downloaded an app in the previous 6 months [17]. E-technologies are also well-accepted by young people as sources of health information. In a recent survey, 39% of young people reported using the Internet to seek information about a mental health problem [18]. An implication of this wide acceptance of e-technologies is that they may offer a medium to improve the well-being of young people by supporting the development of mindfulness [18,19].

The Apple Store now has a staggering 1.4 million apps, more than 35,000 of which are health-related [20]. However, little information is available on the quality or efficacy of these apps beyond user reviews and star ratings [21]. It is imperative that health apps contain high-quality information and have positive effects for users [22].

In particular, while there is growing evidence for the positive effects of face-to-face mindfulness-based training programs, it is unclear if mindfulness apps can provide the same benefits. A search for studies in various databases (ERIC, MEDLINE, PsycINFO, Web of Science, ProQuest) only identified one randomized controlled trial [23] examining the efficacy of a mindfulness training app (Headspace).

The present study conducted a systematic review of mindfulness-based mobile apps, evaluated the quality of these apps using an expert rating scale, and described features of the highest-scoring apps.

## Methods

### Systematic Search

A systematic search of mindfulness-based mobile apps accessible from Australia was conducted in June 2014. The search was conducted using the Google app search function as well as the search feature in the iTunes app store. The Google app search included mindfulness, vipassana, mindful, meditation, and present moment, and excluded hypnosis, hypnotize, weight, magazine, mindmap, mind map, mind-map, and binaural. “Mindfulness” was the only search term used in iTunes, as the search feature was more limited.

Preliminary screening removed irrelevant apps (music/relaxation, happiness, inspirational cards, games, clocks, etc), apps not in English, and those that were not readily accessible. Mindfulness apps that were secular, explicated mindfulness practice, and also had guided mindfulness training were included. Apps that only gave reminders, timers, or guided meditation tracks were excluded, as were apps that cost more than $10 (on the grounds that they were unlikely to be purchased by a large number of users). While guided meditation tracks are a part of mindfulness training, that by itself cannot be justified as mindfulness training as they lack education about mindfulness.

The apps were rated and reviewed in iOS 7 with an iPhone 5s. Each app was tested by at least one author for a minimum of 30 minutes in a real-world setting. The authors were involved in the development of the MARS [24] and had undertaken mindfulness training. Two of the authors had delivered mindfulness training as part of their clinical psychology practice.

### Measures/Rating Tool

The MARS [24] was used to rate app quality. It contains 23 items in 3 sections: classification, app quality, and satisfaction. Each MARS item uses a 5-point scale (1-Inadequate, 2-Poor, 3-Acceptable, 4-Good, 5-Excellent). The classification section is only for descriptive purposes. The 19-item app quality section rates apps on four subscales: engagement, functionality, aesthetics, and information quality. The subjective quality section contains 4 items evaluating the user’s overall satisfaction. The MARS is scored by calculating the mean scores of the app quality subscales and the total mean score. The subjective quality items are scored separately as individual items. The MARS has demonstrated excellent internal consistency (α=0.92) and interrater reliability (ICC=.85) [24]. A second rater reviewed and rated 30% of the apps on the MARS for interrater reliability purposes.

## Results

### Systematic Search

**Figure 1:**
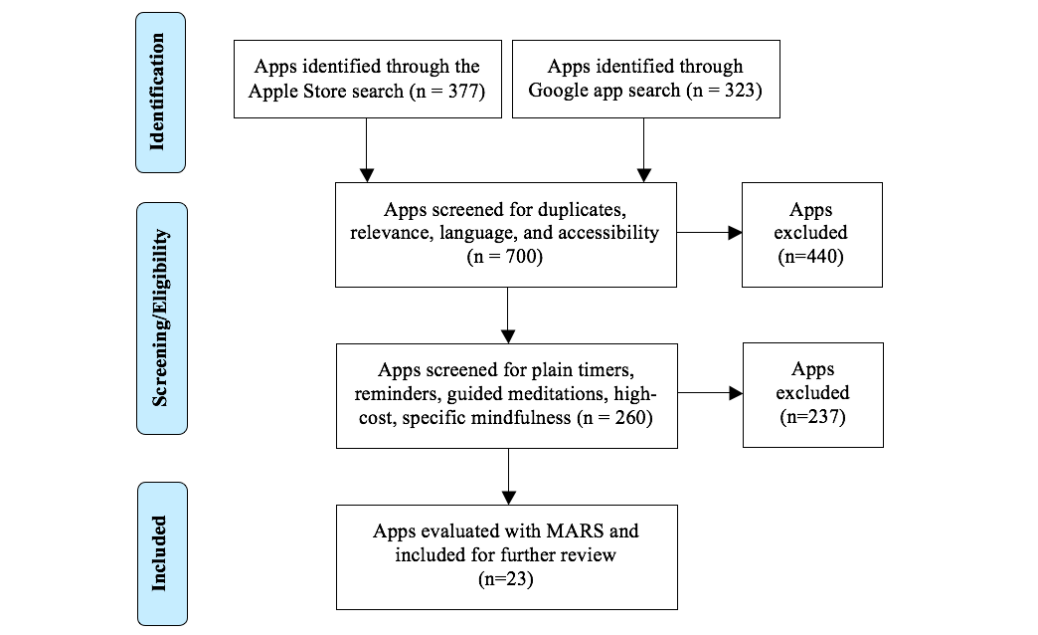
Systematic search for mindfulness apps in Apple store.

The Google and iTunes searches identified 323 and 377 apps, respectively (Figure 1). Excluding duplicates, there were 606 apps. However, 10 were not accessible, 40 were in languages other than English, and 296 were not relevant (ie music/relaxation, happiness, inspirational cards, games, clocks, etc). Of the remaining 260 apps, 23 met the inclusion criteria. Excluded apps comprised those containing timers or reminders (74), guided meditation tracks for common practice or special occasions (129; religious practice/pregnancy/eating/exercise), or information only (37; eBooks/audiobooks/guidelines, without any tools to practice). Nine of the included apps were free and the rest cost between $2.49 and $5.99.

### App Quality

Table 1 shows the subscale and overall scores of apps rated with MARS. It was not possible to rate item 19, which provides a measure of the evidence base for the apps, as a Google Scholar search only identified one efficacy study [23] on one of the included apps (Headspace). Seven apps (30%) were evaluated by two expert MARS raters, and there was an excellent level of interrater reliability (two-way mixed ICC=.84; 95% CI 0.79-0.87).

The Headspace app had the highest average MARS total (4.0) and subscale scores. The next highest were Smiling Mind (3.7), iMindfulness (3.5) and Mindfulness Daily (3.5). Mindfulness Trainer scored the lowest (2.6). The median MARS was 3.2, and all but three of the apps met or beat the minimum acceptability score of 3.0. Satisfaction (the only totally subjective subscale) was not included in the overall score.

### Features of High-Quality Mindfulness Apps

Features of the reviewed apps are summarized in Tables 2 and 3. All contained guided meditations and mindfulness education. They also had at least 2 of the following 9 most common types of guided meditations [25]:

1. Breathing — deep breathing with awareness of the in and out breathes
2. Body scan — awareness of the body focusing on each of the body parts, usually starting from the toes and progressively moving towards the head
3. Sitting meditation — breathing meditation in a sitting posture, with awareness of the body
4. Walking meditation — practicing mindful walking, raising awareness of each movement as we walk slowly
5. Loving kindness meditation — a meditation practice to accept, love and show kindness to oneself and others
6. Thoughts and emotions — acknowledging thoughts and emotions non-judgmentally, as they come and go
7. Mountain meditation — a guided imagery practice, imagining oneself as a mountain and feeling stronger
8. Lake meditation — a guided imagery practice, imagining oneself as a lake, experiencing stillness and peace
9. Three-minute breathing space — a 3-minute guided meditation, with becoming aware in the first minute, gathering and focusing attention in the second minute, and expanding the attention in the third minute.

Almost all apps provided mindful breathing and body-scan exercises. Only one contained all 9 types of guided meditations (Mindfulness Trainer) and few contained loving kindness, lake, and mountain meditations. Buddhify 2 differed from the rest by providing guided meditations to practice in different situations, including exercising, working online, sleeping, and on your work break. The recording quality, voice used, and pace of the delivery of guided meditations varied from app to app.

**Table 1:** MARS Rating.

	App^a^	Engagement	Functionality	Aesthetics	Information^b^	Satisfaction	Overall
1	Headspace^c^	3.8	4.8	4.7	4.0	4.0	4.0
2	Smiling Mind^c^	3.4	4.5	4.3	3.8	4.0	3.7
3	iMindfulness^c^	3.0	4.8	3.7	3.7	2.5	3.5
4	Mindfulness Daily	3.2	4.0	4.0	3.7	3.3	3.5
5	Buddhify 2	3.6	3.8	3.7	3.5	3.8	3.4
6	Complete Mindfulness^c^	3.0	4.0	4.0	3.7	2.8	3.4
7	Mindfulise	3.6	3.5	4.0	3.3	2.5	3.4
8	ACT Coach	3.0	4.0	3.0	3.8	3.5	3.3
9	Rhythm Free	3.4	3.5	4.0	3.2	3.0	3.3
10	Simply8	2.8	3.8	4.0	3.5	2.8	3.3
11	Stop, Breathe & Think	3.2	4.0	3.3	3.3	3.0	3.3
12	Mindfully Me	3.0	4.0	3.3	3.3	2.5	3.2
13	The Meditation App with Michael Stone	3.0	4.0	3.0	3.5	2.5	3.2
14	Meditation without borders^c^	2.6	4.0	3.3	3.5	2.8	3.2
15	Mindfulness Coach	2.8	3.8	3.0	3.7	2.8	3.2
16	The Mindfulness App^a^	3.0	3.8	3.0	3.5	2.5	3.2
17	Take a Chill^c^	3.2	3.5	2.7	3.5	2.5	3.1
18	iMindfulness - On The Go	3.0	3.8	3.0	3.2	2.5	3.1
19	Personal Coach - Mindfulness	3.0	4.0	2.7	3.2	2.5	3.1
20	The Breathing Anchor - Andries J Kroese	2.8	3.8	2.7	3.3	2.5	3.0
21	Mindfulness by Potential Project	2.8	3.5	2.7	3.0	2.0	2.8
22	Cleveland Clinic - Stress Free Now	2.4	3.8	2.7	3.0	2.5	2.8
23	Mindfulness Trainer	2.2	3.3	2.3	3.0	1.8	2.6

^a^ The rated versions (Multimedia appendix 1) of the apps may not be available in the App Store at the time of publication, as they may be replaced by newer versions.

^b^ The information quality score excluded Item 19 of the MARS.

^c^ Rated by two raters for interrater reliability purposes.

**Table 2:** Summary of mindfulness-based apps features.

#	App	Timer	Reminders	Mood assessments	Tracking	PB Practice^a^
1	Headspace	✓	✓		✓	✓
2	Smiling Mind	✓	✓	✓	✓	✓
3	iMindfulness	✓	✓		✓	
4	Mindfulness Daily	✓	✓	✓	✓	✓
5	Buddhify 2	✓			✓	
6	Complete Mindfulness					
7	Mindfulise	✓			✓	
8	ACT Coach				✓	
9	Rhythm Free	✓	✓		✓	
10	Simply8	✓	✓			✓
11	Stop, Breathe & Think			✓	✓	
12	Mindfully Me	✓	✓		✓	
13	The Meditation App with Michael Stone	✓	✓		✓	
14	Meditation without borders					✓
15	Mindfulness Coach	✓	✓		✓	
16	The Mindfulness App	✓	✓		✓	
17	Take a Chill	✓	✓		✓	
18	iMindfulness On The Go	✓	✓		✓	
19	Personal Coach - Mindfulness	✓	✓			
20	The Breathing Anchor - Andries J Kroese	✓	✓		✓	
21	Mindfulness by Potential Project	✓	✓		✓	
22	Cleveland Clinic - Stress Free Now					
23	Mindfulness Trainer					

^a^Program-based practice

**Table 3:** Summary of mindfulness-based app features.

#	App	App community	Social Media	In-app Purchase	Cost
1	Headspace	✓	✓	✓	Free
2	Smiling Mind		✓		Free
3	iMindfulness			✓	$2.49
4	Mindfulness Daily		✓	✓	Free
5	Buddhify 2			✓	$3.79
6	Complete Mindfulness				$2.49
7	Mindfulise				$3.79
8	ACT Coach				Free
9	Rhythm Free		✓	✓	Free
10	Simply8		✓		$3.79
11	Stop, Breathe & Think		✓		Free
12	Mindfully Me		✓		Free
13	The Meditation App with Michael Stone		✓		$3.79
14	Meditation without borders		✓		$5.99
15	Mindfulness Coach				Free
16	The Mindfulness App		✓	✓	$2.49
17	Take a Chill		✓	✓	$2.49
18	iMindfulness On The Go			✓	$2.49
19	Personal Coach - Mindfulness				$2.49
20	The Breathing Anchor - Andries J Kroese				$2.49
21	Mindfulness by Potential Project				$2.49
22	Cleveland Clinic - Stress Free Now				Free
23	Mindfulness Trainer				$3.79

The majority of apps contained timers and provided reminders. Seven did not have a timer (ACT Coach, Complete Mindfulness, Stop, Breathe & Think, Meditation without Borders, MindKind Now, Cleveland Clinic - Stress Free Now, Mindfulness Trainer) and nine did not have reminders (ACT Coach, Buddhify 2, Cleveland Clinic - Stress Free Now, Complete Mindfulness, Meditation without Borders, Mindfulise, Mindfulness Trainer, MindKind Now, Stop, Breathe & Think).

Five apps provided progressive/program-based mindfulness training (Headspace, Smiling Mind, Mindfulness Daily, Simply8 and Meditation without Borders). Headspace provided free access to a 10-day program, Take 10, which has 10 guided meditation sessions of approximately 10 minutes each. Completing a session unlocked the next meditation track. Smiling Mind had a 10-week program for different age groups. The introductory session at the start of each week explored breath, sounds, tastes, etc. The user was advised to practice mindfulness and relevant take-home activities with the assistance of the app. Simply8 was a 3-week program with 8 minutes of guided meditation every day under the themes of calm, clear, and aware (focusing on one theme each week). Mindfulness Daily provided short mindfulness exercises for 21 days. The user can also access guided meditations such as body scan, kindness, and awareness any time. Meditation without Borders was a 4-week program advising the users to practice guided meditations for at least 20 minutes per day.

While most apps provided exclusive texts and videos explaining the concepts of mindfulness, some apps relied on guided meditation tracks to educate the user. Take A Chill referred to relevant websites and did not provide much mindfulness education within the app. Few apps (eg ACT Coach, Complete Mindfulness) provided comprehensive text-based education. Headspace used video infographics to explain the concepts. Two of the apps (Mindfulness by Potential Project and iMindfulness) mentioned the 7 attitudes for mindfulness training, otherwise known as the essential pillars of Mindfulness-Based Stress Reduction (MBSR) practice [25].

Twelve apps provided an option to share the user’s experience in social networks such as Facebook and Twitter (Headspace, Meditacious, Meditation without Borders, Mindfully Me, Mindfulness Daily, Rhythm Free, Simply8, Smiling Mind, Stop, Breathe & Think, Take A Chill, The Meditation App with Michael Stone, The Mindfulness App). Headspace and Meditacious also had an app community. Eight apps provided in-app purchase that included additional guided meditation tracks (Take a Chill, iMindfulness On The Go, Headspace, Mindfulness Daily, The Mindfulness App, iMindfulness, Buddhify 2, and Rhythm Free, which also provided reminders)

##  Discussion

### Principal Findings

Though the search for mindfulness apps identified 606 apps, excluding duplicates, only 23 provided mindfulness training. Timers, reminders, meditation, relaxation, or reference apps can assist in mindfulness practice, but categorizing them as mindfulness apps is inappropriate [26].

Mindfulness is much more than meditation, a breathing exercise, or a relaxation technique. Meditation is a practice that aids development of mindfulness [27,28]. Breathing is used as an exercise in the practice of mindfulness and relaxation can be an outcome. Contemplative practices (breathing, sitting, walking meditations), understanding emergent bodily and mental experiences, and withdrawing from habitual experiential avoidance form part of mindfulness training in mindfulness-based interventions such as MBSR and Mindfulness-Based Cognitive Therapy [29]. A mindfulness app should clearly explain the philosophy and practice of mindfulness and address common misconceptions. An app without mindfulness education may be beneficial if this information has been provided as part of face-to-face mindfulness training. However, a stand-alone mindfulness app should educate the user on mindfulness. All of the apps included in the review explain the concept of mindfulness at varying levels. Some (eg Headspace, Smiling Mind) employed interesting visual modes of explanation.

Mindfulness is a habit and a mind-training skill that requires regular practice and sustained effort to be effective [3,30-32]. This is a challenge for both face-to-face and app-based mindfulness training. Mindfulness apps provide 24/7 access to mindfulness-based practice. Interactive mobile applications and aesthetically pleasing and well-designed apps are likely to be more effective in engaging the user in regular mindfulness practice [33,34]. Headspace, Mindfulise, Buddhify 2 and Smiling Mind exceeded the minimum acceptable level score (3.0) on the MARS engagement subscale. These apps had high-quality graphics, simple and easy-to-use interfaces, and soothing voices for the guided meditation tracks. Headspace used short video infographics that complemented the guided meditation tracks. Unlike most apps that used a linear menu style, Buddhify 2 used an interesting collapsible circular menu to choose the meditation tracks. The low median score of the reviewed apps on the MARS engagement subscale, highlights the need to focus on engagement and motivation during the design process.

Participation in an app community can help motivate users to engage in healthy activities [35]. A supportive app community can help users share and discuss their mindfulness experiences and the challenges of regular practice. This could potentially complement or substitute for the support provided in face-to-face mindfulness training. While nearly 50% of the reviewed apps provided social network sharing, only Headspace and Meditacious incorporated app community support. Research is required to determine the impact of sharing in social media and participating in a supportive app community on the frequency of mindfulness-based practice.

Assessing the quality of an app, especially a health intervention app, is an essential step before evaluating its efficacy [36]. The 23 mindfulness apps reviewed in this study had a median objective quality MARS score of 3.2. This suggests the apps had an overall acceptable level of quality. However, the low median engagement and moderate median aesthetics and information subscale scores highlight potential target areas for improvement.

### Strengths and Limitations

This study is one of the first to review mindfulness-based mobile apps and evaluate their quality using a new multidimensional expert rating scale. The MARS provides a reliable measure of app quality on four objective subscales (engagement, functionality, visual aesthetics, information quality) and one subjective scale. Only the objective quality scales are included in the total app quality score. Expert ratings on 30% of the reviewed apps had a high level interrater reliability in the current study. However, while the MARS can be used to provide an evaluation of the quality of existing apps, this cannot replace the use of rigorous user-centered design and evidence-based practice in the design of health behavior apps.

The current review was limited to iPhone iOS apps, indicating future research is required to review and rate the quality of mindfulness apps developed for Android and other app platforms. Future research is also required to assess the quality of mindfulness training and individual guided meditation tracks contained in the apps, as there is currently no gold standard for how mindfulness is best conceptualized or practiced.

### Future Research

mHealth is fast becoming an essential component of global health care [37]. The majority of mHealth apps developed to date have focused on physical health and lifestyle domains rather than mental health [38,39]. While an increasing number of mindfulness apps are being developed, the current evidence base is limited to one trial examining the efficacy of the Headspace app [23]. Future research is needed to determine and compare the efficacy of mindfulness apps in randomized controlled trials.

### Conclusions

Only 4% of the 700 apps identified in our search provided mindfulness training and education. Though many apps claimed to be mindfulness apps, most of them were not. While the reviewed apps scored an acceptable median MARS score, very few scored high, indicating that the quality of the apps can be improved. The lack of evidence for the effectiveness of mindfulness apps needs to be addressed.

## Appendix

Multimedia Appendix 1 Mindfulness-based iPhone apps.
